# Widely targeted metabolomic and transcriptomic analyses of the effects of blue polarized light and ordinary light on *Dendrobium officinale*

**DOI:** 10.3389/fpls.2026.1717555

**Published:** 2026-02-24

**Authors:** Hansheng Li, Beibei Chen, Ruichen Li, Haibin Wang, Jin Sun, Gang Sun

**Affiliations:** 1 Fujian Provincial Key Laboratory of Bamboo Resources Development and Utilization, Sanming University, Sanming, China; 2 College of Horticulture, Nanjing Agricultural University, Nanjing, China; 3 College of Resources and Chemical Engineering, Sanming University, Sanming, China

**Keywords:** blue polarized light, dendrobium officinale, ordinary light, transcriptomic, widely targeted metabolomic

## Abstract

**Introduction:**

*Dendrobium officinale*, an orchid native to China, benefits the stomach, moistens the lungs, and enhances immunity. Light affects the synthesis of functional metabolites in *D. officinale*. The luminescence mechanism of polarized light differs from that of ordinary light sources and has different effects on plants.

**Methods:**

In this study, different light treatments—white (W), blue (B), and blue polarized (BP) light—were applied to *D. officinale*. In comparison to ordinary light sources, blue polarized light significantly altered the stem color, making it reddish. RNA-seq and targeted metabolomics analysis were used to investigate the mechanisms underlying the effects of the different light treatments on *D. officinale*.

**Results:**

The results revealed that 2448, 2065, and 2763 genes and 1190, 812, and 958 metabolites were differentially expressed in the BP-B, BP-W and W-B comparisons, respectively. GO and KEGG analyses of the DEGs revealed that the most significant differences between *D. officinale* under polarized light and ordinary light sources were in pathways related to microtubules, UDP-glycosyltransferase activity and microtubule binding. Metabolome analysis revealed that the expression of melanoside A, 1-methyl-L-histidine, and rhapontigenin 3'-O-glucoside were the SCMs most strongly affected by blue polarized light. The use of bidirectional orthogonal partial least squares analysis revealed a significant transcriptome-metabolome correlation in the BP-W and BP-B comparisons. Joint analysis of DEGs and SCMs revealed significant differences between polarized and ordinary light sources, mainly in terms of plant hormone signal transduction, zeatin biosynthesis, phenylpropanoid biosynthesis, and flavonoid biosynthesis.

**Discussion:**

These results highlight that blue polarized light enhances flavonoid and phenylpropanoid metabolism, offering a strategy for improved secondary metabolite yield in *D. officinale* cultivation.

## 1 Introduction

*D. officinale* Kimura et Migo is an herbaceous medicinal plant belonging to the Dendrobium genus in the family Orchidaceae. It is among the traditional prized Chinese medicinal materials and ranks first among the “Nine Immortal Herbs” in China (Chen X. et al., 2025). Its various effects include generating fluids, moistening the lungs, nourishing the five organs, and enhancing immunity. Modern pharmacological research has shown that its main components include polysaccharides, flavonoids, and dibenzyl compounds, which exhibit pharmacological effects such as antioxidant, hypoglycemic, cholesterol lowering, and cardiovascular system protection effects (Zeng et al., 2024; Wu et al., 2025). *D. officinale* has outstanding medicinal value and a significant economic role, and there has been a continuous increase in consumer demand for this plant.

Researchers have increased the functional metabolite content in *D. officinale* through different methods, among which light regulation is important (Yang et al., 2021). Ordinary light sources include natural light and artificial sources and refers to light sources with disordered electromagnetic waves, that is, waves that are randomly oriented in all directions. In contrast, the electromagnetic waves of polarized light sources exhibit specific regularity: they are oriented only in a specific direction (linearly polarized light) or rotate according to a certain pattern (circularly polarized light or elliptically polarized light) (Agrawal, 2025). Polarized light sources have completely different luminescence mechanisms than ordinary light sources do, which not only effectively solves the problems associated with ordinary light sources but also may be used to explore new mechanisms underlying the effects of light on plant metabolism. There is currently limited research on the effects of polarized light on plants. However, previous studies have shown that blue polarized light and green polarized light have significantly different effects than nonpolarized light on growth and development in maize (Kulchin et al., 2024).

Progress has been made in understanding the molecular mechanism underlying the regulation of plant functional metabolites through the use of ordinary light sources. Ordinary light sources can regulate the accumulation of functional metabolites in plants directly or indirectly through metabolic pathways and structural genes (Zhang et al., 2017).

Chalcone synthase (CHS) is the first key enzyme in the flavonoid biosynthesis pathway. It catalyzes the binding of three molecules of malonyl-CoA and one molecule of coumaroyl-CoA to produce one molecule of chalcone, which is also the basis for the synthesis of other flavonoids (Zhang et al., 2017). Wu et al. (2020) conducted a study on the heterologous transformation of tobacco with TrCHS from *Trifolium repens* and reported that both the expression level of TrCHS and the total flavonoid content significantly increased. Transcription factors can regulate gene expression levels by indirectly or directly interacting with the promoters of metabolic pathway structural genes, thereby playing a role in regulating the synthesis of functional metabolites in plants (Wen et al., 2020). Illumination cultivation of cabbage overexpressing *AtMYB*12 significantly increased the total phenolic acid and flavonoid contents of cabbage, which is closely related to the activation of the flavonoid metabolic pathway in the light response by the *AtMYB*12 gene (Meng et al., 2020). Previous studies have elucidated several signal transduction pathways among regulating plant functional metabolites through ordinary light sources, such as the COP1/SPA pathway, CIB1 pathway, and PIF pathway (Yang et al., 2017; Zhang et al., 2021; Favero, 2020). For example, PIFs can transduce light signals downstream of phytochrome and cryptochrome receptors and integrate signals such as those related to hormones, sugar metabolism, and circadian rhythms with environmental pathway signals such as temperature, light quality, light intensity, and photoperiod to synergistically participate in plant morphogenesis, shading avoidance responses, anthocyanin synthesis, reactive oxygen species, and chlorophyll and carotenoid pigment synthesis, as well as other transcriptional responses (Monte, 2020). However, the molecular mechanism by which polarized light sources affect plant growth, development, and functional metabolites has not been reported to date.

In this study, transcriptome sequencing and plant-wide target metabolite data from plants exposed to white light, blue light, and blue polarized light were analyzed and compared with the goal of exploring the molecular mechanisms underlying the effects of ordinary light sources and polarized light sources on growth, development, and functional metabolism in *D. officinale*. We also aimed to identify specific genes that regulate the functional metabolites of *D. officinale* under blue polarized light. These results provide new insights into the high-yield production of medicinal components in *D. officinale*.

## 2 Results

### 2.1 Growth state of *D. officinale* under different light treatments

**Figure 1:**
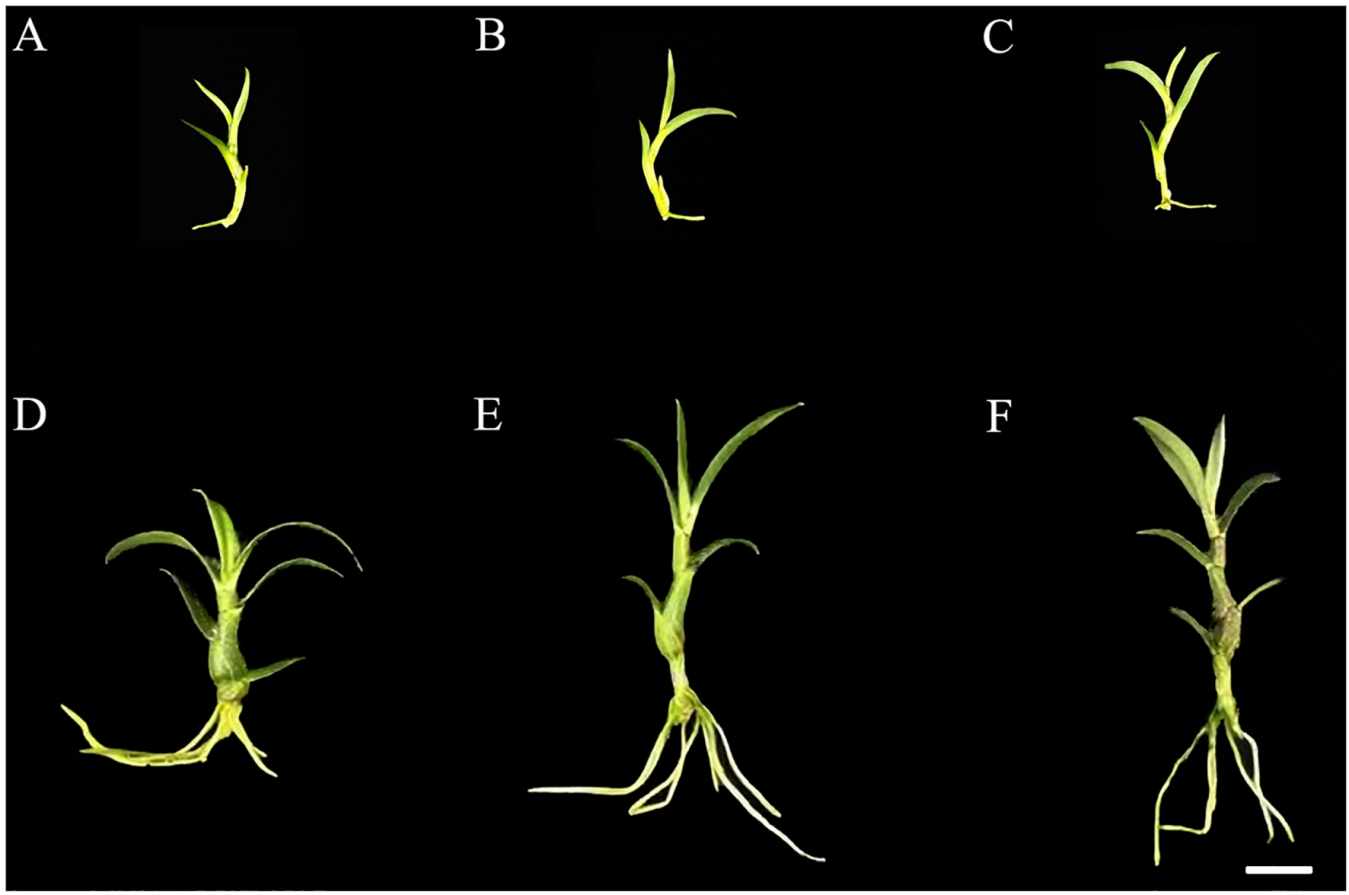
Phenotype of *D. officinale* under different light treatments. **(A–C)** show the phenotypes before white, blue and blue polarized light treatments, respectively. **(D–F)** show the phenotypes after white, blue and blue polarized light treatments, respectively. Bars=10 mm.

Polarized light can affect the phenotype of *D. officinale*. In our study, the largest blade area was found upon exposure to white light, followed by blue light, and finally blue polarized light. The height of the plants under blue polarized light and blue light did not differ, while those under white light were the shortest. The stems under blue polarized light and blue light were relatively consistent, while the stems under white light were the thickest, and the stem color under blue polarized light was more red in color (**Figure 1**, **Supplementary Tables S1, S2**). Therefore, the phenotypes of *D. officinale* differ significantly between polarized light sources and ordinary light sources.

### 2.2 Sequencing and assembly of transcriptomic data

To investigate the effects of polarized light on functional metabolite-related genes in *D. officinale*, 9 mRNA libraries were constructed, and sequencing was conducted. After the adapter sequences were removed, the number of reads generated from the RNA-seq data of *D. officinale* under different light treatments ranged from 40062924 to 50892606. A total of 91.24–92.61% of the clean reads could be fully aligned to the reference genome of *D. officinale*, and 87.40–89.80% of the clean reads could be aligned to a single locus of the reference genome of *D. officinale*, indicating a high degree of matching between the sequencing reads and the reference genome of *D. officinale*. The Q30 values of the *D. officinale* samples were greater than 97.32%, indicating that the transcriptome sequencing data of *D. officinale* in this study are highly reliable (**Supplementary Table S3**).

### 2.3 Analysis of differentially expressed genes under different light treatments

**Figure 2:**
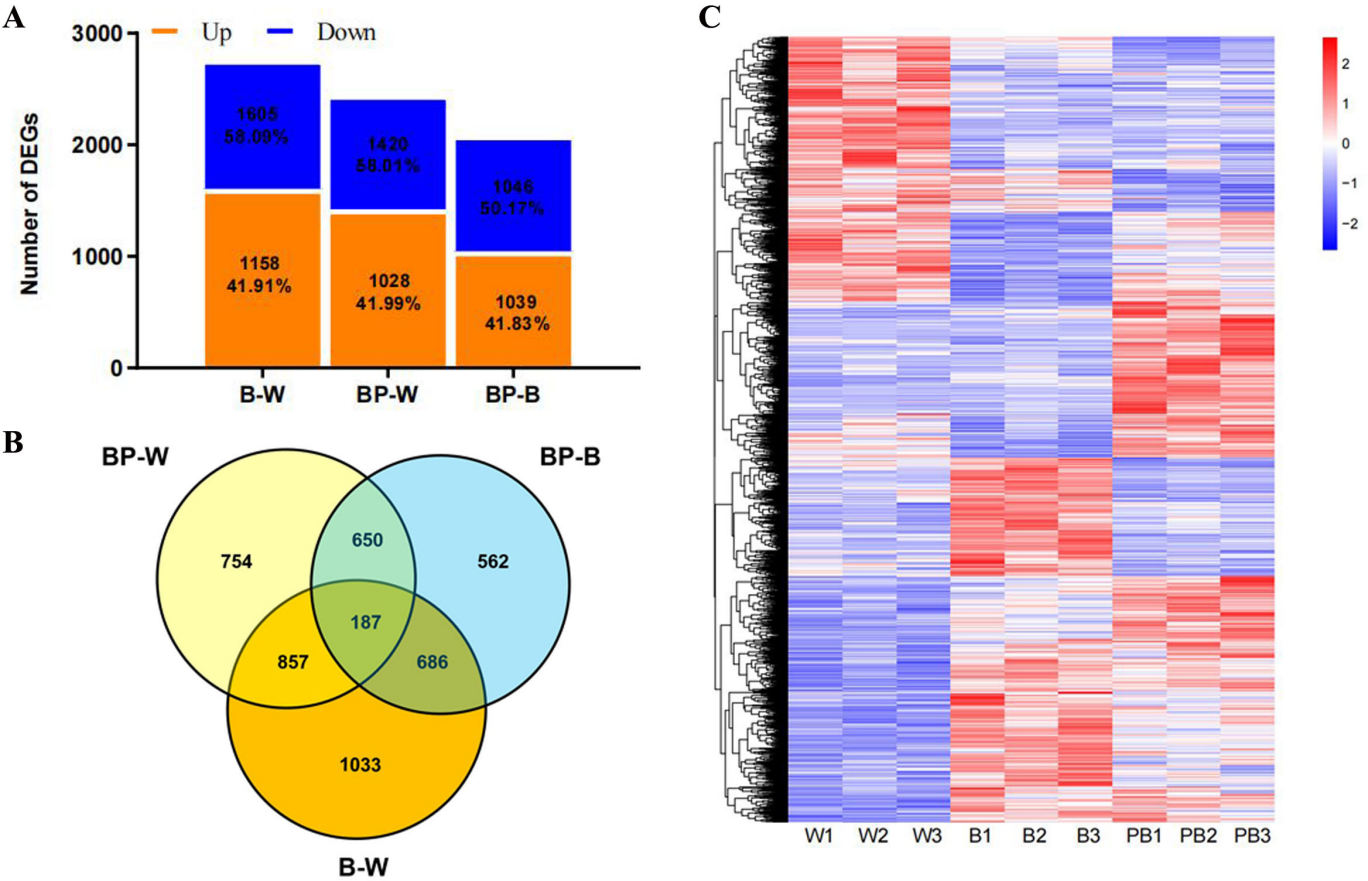
Differentially expressed genes under the different light treatments. **(A)** Number of genes whose expression was up- and downregulated in each library. **(B)** Venn diagram showing the numbers of unique and commonly regulated genes identified from the B-W, BP-W and BP-B comparisons. **(C)** DEGs in response to different light treatments.

In this study, the transcriptome sequencing results were analyzed, and 2835 new genes were identified, 1378 of which were functionally annotated. 4729 of which were differentially expressed genes (DEGs) (**Supplementary Table S4**). To investigate the expression status of genes in *D. officinale*, all genes were divided into upregulated and downregulated categories, and the results revealed that most genes were upregulated (**Figure 2A**). To study the differential gene expression of *D. officinale*, Venn diagrams were constructed to identify overlap in the DEGs from different between-group comparisons. A total of 2448 DEGs were identified in the BP-W comparison, 2065 DEGs were identified in the BP-B comparison, and 2763 DEGs were identified in the B-W comparison. A total of 187 genes were changed in all three comparisons (BP-W, BP-B, and B-W); 857 genes were changed in both the BP-W and B-W comparisons; 686 genes were changed in both the BP-B and B-W comparisons; and 650 genes were changed in both the BP-W and BP-B comparisons. A total of 754 genes were specifically changed in the BP-W comparison, 562 genes were specifically changed in the BP-B comparison, and 1033 genes were specifically changed in the B-W comparison (**Figure 2B**). Clustering analysis was then performed to classify the 4729 differentially expressed genes into six expression patterns under different light treatments (**Figure 2C**; **Supplementary Table S5**).

### 2.4 GO enrichment analysis of differentially expressed genes

**Figure 3:**
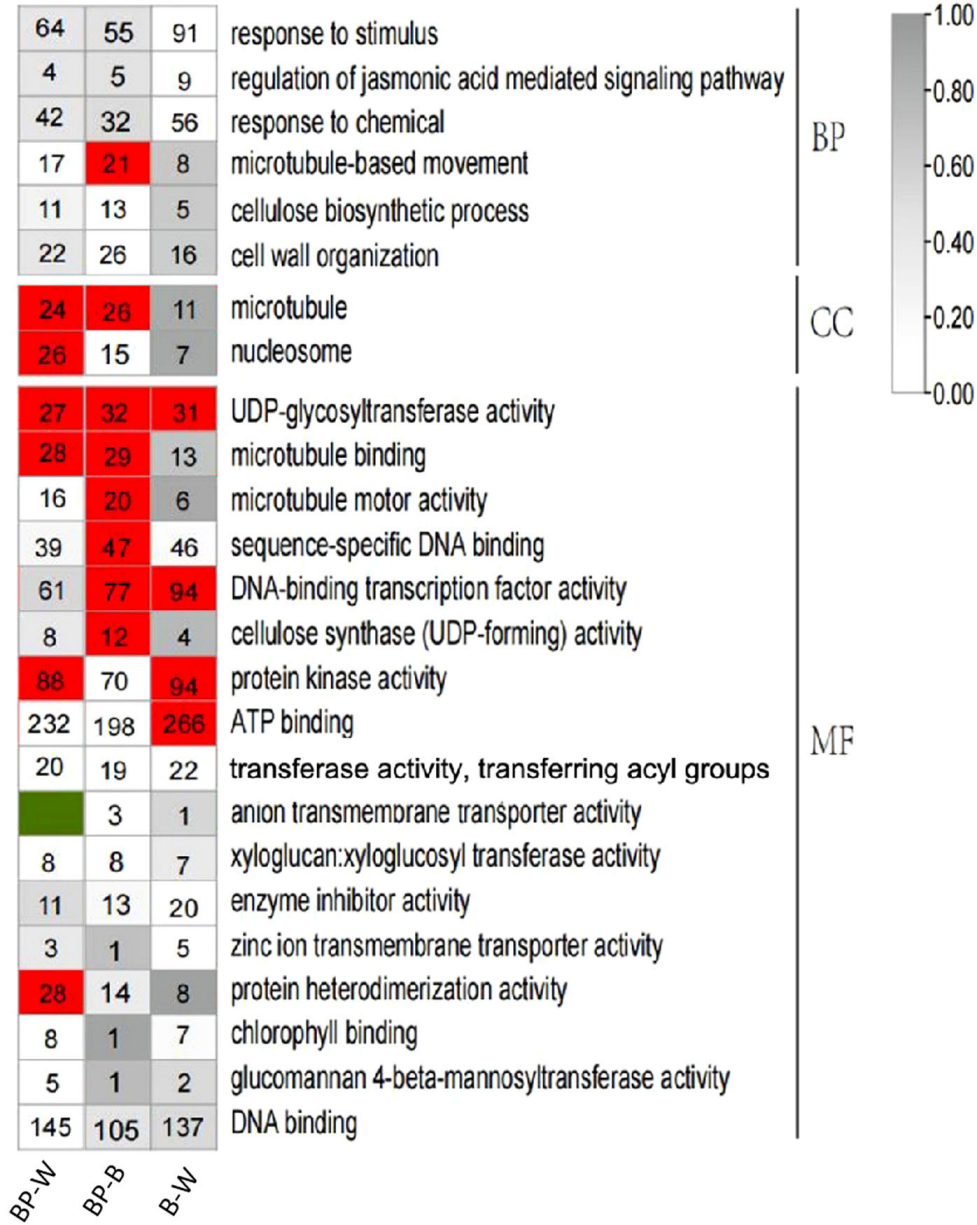
GO enrichment analysis of differentially expressed genes. The number in the color block is the number of differentially expressed genes; a red color block indicates P ≤ 0.01.

To further understand the effect of polarized light on *D. officinale*, a GO enrichment analysis was performed on the DEGs associated with the BP-W, BP-B, and B-W comparisons (**Figure 3**).

Among the BP-W DEGs, the significantly correlated cellular component terms included mainly microtubules and nucleosomes. The significantly related molecular function terms included mainly UDP-glycosyltransferase activity, microtubule binding, protein kinase activity and protein heterodimerization activity.

Among the BP-B DEGs, the significantly correlated biological processes included mainly microtubule-based movement. The significantly related cellular component terms included mainly microtubules. The significantly correlated molecular function terms included mainly UDP-glycosyltransferase activity, microtubule binding, microtubule motor activity, sequence-specific DNA binding, DNA-binding transcription factor activity and cellulose synthase (UDP-forming) activity.

### 2.5 Functional classification of the light-induced conserved genes in *D. officinale*

**Figure 4:**
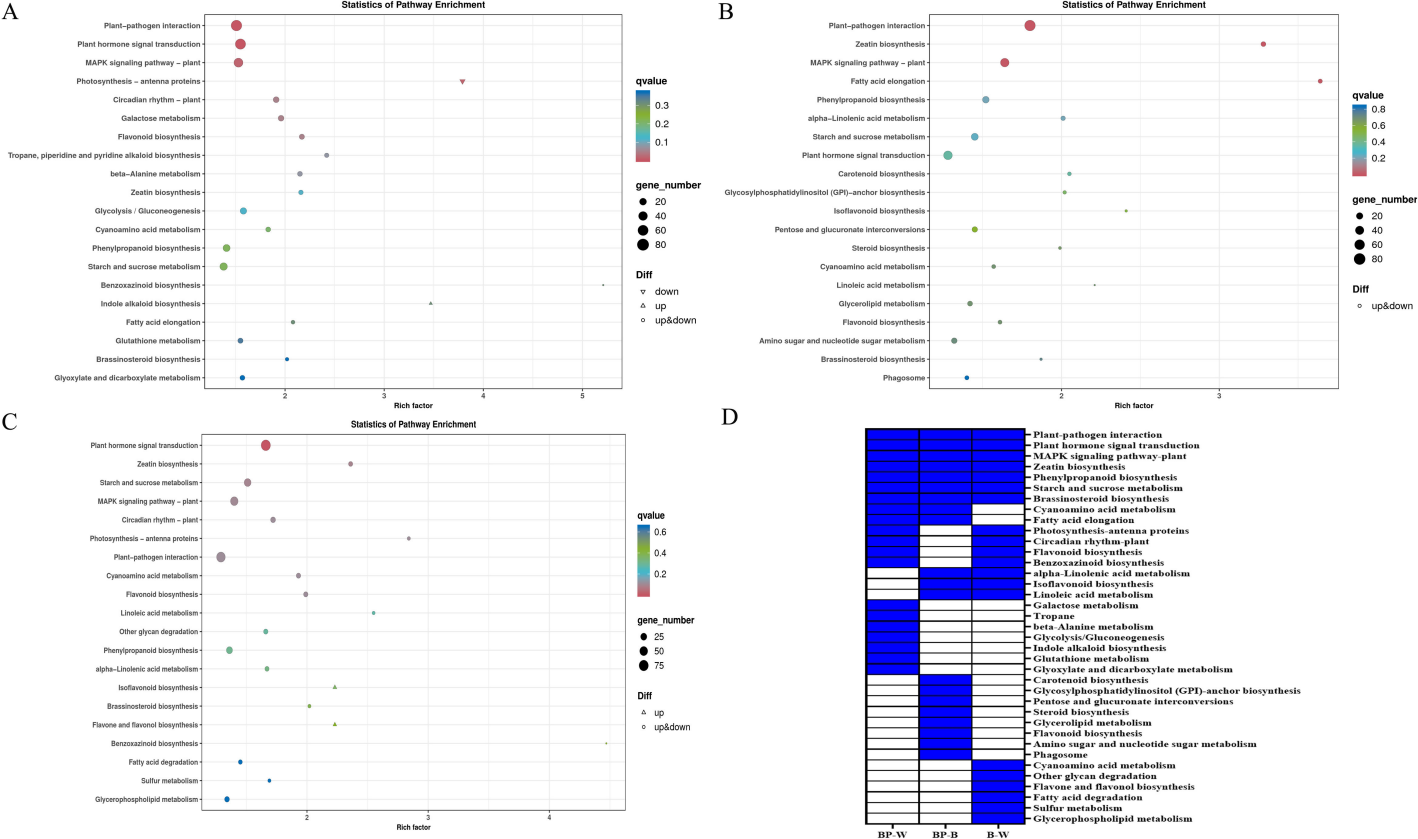
KEGG enrichment analysis of DEGs in *D*. *officinale* under different lighting treatments. **(A)** BP-W. **(B)** BP-B. **(C)** B-W. **(D)** The top 20 enriched KEGG pathways among the DEGs in the three groups. The blue color indicates that the comparison contains the pathway, and the white color indicates that the comparison does not contain the pathway.

We performed MapMan KEGG enrichment analyses for the DEGs (**Figure 4**). The top 5 pathways enriched among the BP-W DEGs included benzoxazinoid biosynthesis; photosynthesis-antenna proteins; indole alkaloid biosynthesis; tropane, piperidine and pyridine alkaloid biosynthesis; and flavonoid biosynthesis (**Figure 4A**). The top 5 pathways enriched among the BP-B DEGs included fatty acid elongation, zeatin biosynthesis, isoflavonoid biosynthesis, linoleic acid metabolism and carotenoid biosynthesis (**Figure 4B**). The top 5 pathways enriched among the B-W DEGs included linoleic acid metabolism, photosynthesis-antenna proteins, zeatin biosynthesis, isoflavonoid biosynthesis, and flavone and flavonol biosynthesis (**Figure 4C**).

In this study, the top 20 pathways enriched among each of the three comparisons (BP-W, BP-B, and B-W) were analyzed. Some pathways were found within the top 20 pathways in all three sets of DEGs (**Figure 4D**); for example, plant–pathogen interaction, plant hormone signal transduction, MAPK signaling pathway-plant, zeatin biosynthesis, phenylpropanoid biosynthesis, starch and sucrose metabolism and brassinosterol biosynthesis were significantly enriched in all three groups (BP-W, BP-B, and B-W).

Some pathways were found within the top 20 pathways in two sets of DEGs, such as cyanoacetic acid metabolism and fatty acid elongation, which were enriched in the BP-W and BP-B combinations (**Figure 4D**), indicating that blue polarized light may have a significant effect on these pathways.

Some pathways, such as galactose metabolism, tropane, piperidine and pyridine alkaloid biosynthesis, beta-alanine metabolism, glycolysis/gluconeogenesis, indole alkaloid biosynthesis, glutathione metabolism, and glyoxylate and dicarboxylate metabolism, were in the top 20 pathways enriched in the BP-W DEGs. Carotenoid biosynthesis, glycosylphosphatidylinositol (GPI)-anchor biosynthesis, pentose and glucuronate interconversions, steroid biosynthesis, glycerolipid metabolism, flavonoid biosynthesis, amino sugar and nucleotide sugar metabolism, and the phagosome were enriched among the top 20 pathways in the BP-B DEGs. These results indicate that blue polarized light may have a significant effect on these pathways.

### 2.6 Changes in metabolites under different lighting treatments

**Figure 5:**
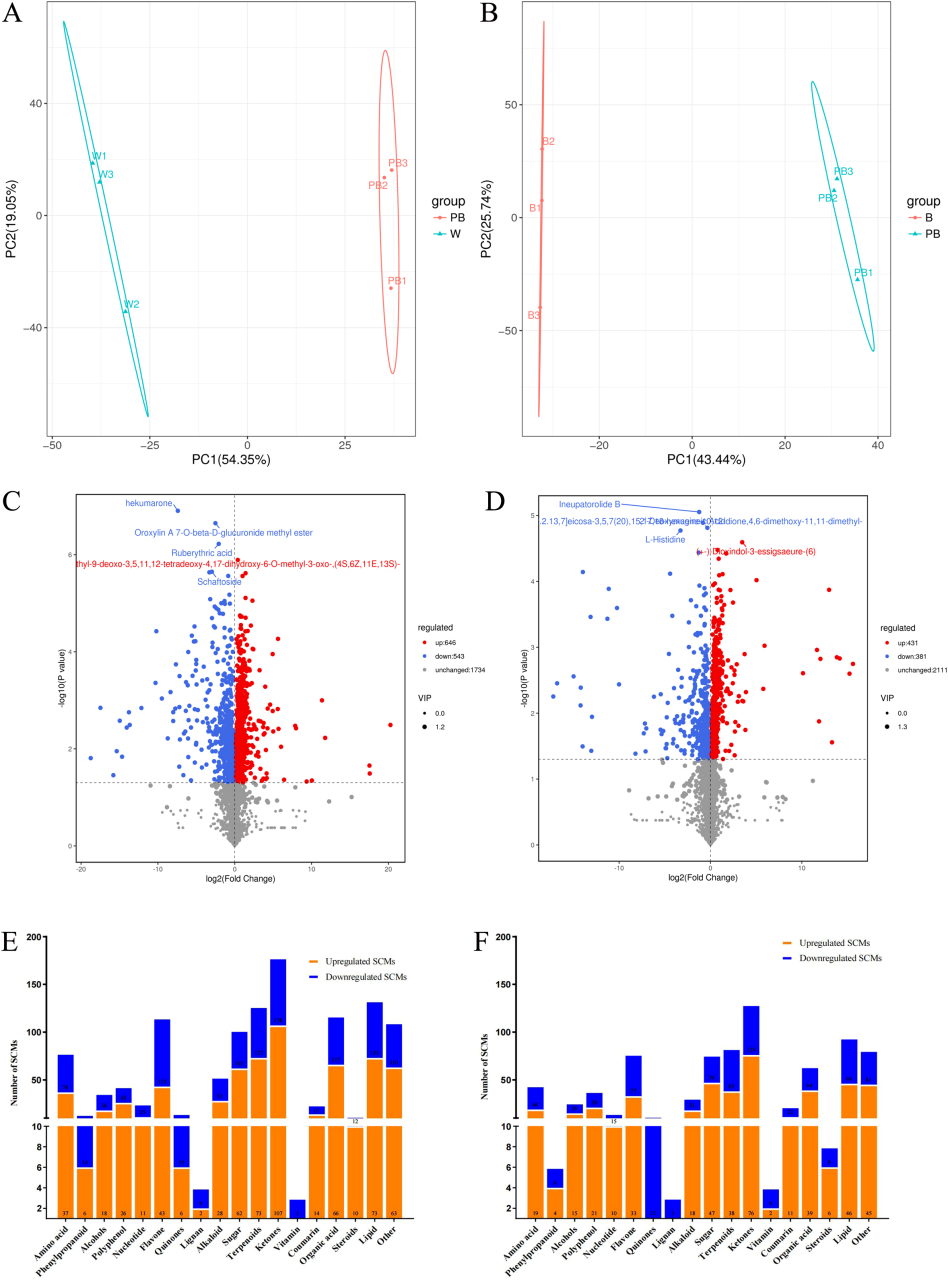
Multivariate statistical analysis of metabolites from BP-W and BP-B. **(A, B)** represent PCA score plots of metabolites between the young shoots of BP-W and BP-B, respectively. **(C, D)** show volcano plots of metabolites between the young shoots of BP-W and BP-B, respectively. **(E, F)** represent the number of SCMs.

OPLS-DA revealed that the metabolite profiles in the BP, B, and W treatments significantly differed from one another (**Figures 5A, B**). On the basis of the VIP values and fold changes of the metabolites, a total of 1190 differentially abundant metabolites (DAMs) in the leaves of *D. officinale* were detected and quantified in the BP-W comparison; 812 metabolites were detected and quantified in the BP-B comparison. A volcano plot revealed that the number of upregulated metabolites was significantly greater than the number of downregulated metabolites (**Figures 5C, D**). These specific metabolites (SCMs) can be classified into 18 categories. Notably, the BL-W SCMs included more upregulated SCMs than downregulated SCMs, mainly polyphenols, alkaloids, sugars, terpenes, ketones, aldehydes, esters, coumarins, organic acids, steroids, and lipids. Among the BL-B SCMs, the number of upregulated SCMs was also greater than the number of downregulated SCMs and included mainly alcohols, polyphenols, nucleotides, alkaloids, sugars, ketones, aldehydes, esters, organic acids, and steroids (**Figures 5E, F**).

In the BP-W combination, 5 out of the top 10 metabolites among the upregulated SCMs were flavonoids, 2 out of the downregulated SCMs were flavonoids, 2 were polyphenols, and 2 were lipids. The top 3 metabolites among the upregulated SCMs were melanoside A, lucenin II, and 2’-O-β-L-gallientin, all of which are flavonoids. The top 3 metabolites among the downregulated SCMs were 13,21-dihydroeurycomanone, isovitexin 2’’-O-arabinoside, and rhapontigenin 3’-O-glucoside (**Table 1**).

**Table 1:** The top 10 metabolic pathways among the up and down regulated SCMs in BP-W.

ID	Metabolite	Class I	W_Mean	PB_Mean	log2FC	Regulated
POS_q297	Meloside A	Flavonoids	10.07	12675442.71	20.26	up
POS_q280	Lucenin II	Flavonoids	10.07	1956137.81	17.57	up
POS_q30	2’’-O-β-L-galorientin	Flavonoids	10.07	1911055.75	17.53	up
NEG_q12	2,4-Dihydroxybenzoic acid	Polyphenols	10.07	34977.23	11.76	up
NEG_q102	Crotanecine	Alkaloids	10.07	26344.20	11.35	up
NEG_q87	Batatasin Iii	Polyphenols	10.07	10638.32	10.05	up
POS_q222	Higenamine (hydrochloride)	Alkaloids	10.07	6616.98	9.36	up
NEG_q55	7-Hydroxyflavanone	Flavonoids	10.07	2535.61	7.98	up
NEG_t911	Vitexin 2’’-O-(4’’’-O-acetyl)rhamnoside	Flavonoids	10.07	2352.27	7.87	up
POS_t608	(1R,2S,11S,14S,15R,20S)-15-(hydroxymethyl)-1,11,19,19-tetramethyl-10- oxapentacyclo[12.8.0.02,11.04,9. 015,20]docosa-4(9),5,7-trien-6-ol	Coumarins	10.07	862.70	6.42	up
POS_q74	(+)-Afzelechin	Flavonoids	45784.32	10.07	-12.15	down
POS_q345	Phendimetrazine	Others	130905.63	10.07	-13.67	down
NEG_q349	α-Linolenic acid	Lipid	140805.71	10.07	-13.77	down
POS_q354	Pilocarpine	Alkaloids	171699.29	10.07	-14.06	down
POS_q160	D-Panthenol	Lipid	261278.44	10.07	-14.66	down
NEG_q3	1-(2,3,4-Trihydroxyphenyl)ethanone	Ketones, aldehydes, esters	321596.65	10.07	-14.96	down
NEG_q277	Piceatannol 3’-O-glucoside	Polyphenols	422516.03	10.07	-15.36	down
POS_q4	13,21-Dihydroeurycomanone	Terpenoids	573729.35	10.07	-15.80	down
POS_q239	Isovitexin 2’’-O-arabinoside	Flavonoids	1838494.09	10.07	-17.48	down
NEG_q294	Rhapontigenin 3’-O-glucoside	Polyphenols	4417159.10	10.07	-18.74	down

In the BP-B combination, 3 out of the top 10 metabolites among the upregulated SCMs were flavonoids, 2 out of the downregulated SCMs were flavonoids, 2 were polyphenols, and 2 were lipids. The top 3 metabolites among the upregulated SCMs were 1-methyl-L-histidine, genistein 7,4’-di-O-beta-D-glucopyranoside, and 1,3-dicaffeoylquinic acid. The top 3 metabolites among the downregulated SCMs were 13,21-dihydroeurycomanone, isovitexin 2’’-O-arabinoside, and rhapontigenin 3’-O-glucoside, which were the same as those associated with the BP-W combination (**Table 2**).

**Table 2:** The top 10 metabolic pathways among the up and down regulated SCMs in BP-B.

ID	Metabolite	Class I	B_Mean	PB_Mean	log2FC	Regulated
POS_q15	1-Methyl-L-histidine	Amino acids	10.07	501893.29	15.61	up
POS_q194	Genistein 7,4’-Di-O-Beta-D-glucopyranoside	Flavonoids	10.07	385613.74	15.23	up
NEG_q4	1,3-Dicaffeoylquinic acid	Organic acid	10.07	183082.05	14.15	up
NEG_q235	Methylnissolin-3-O-glucoside	Flavonoids	10.07	147745.98	13.84	up
POS_q339	Palmatine (chloride)	Alkaloids	10.07	102209.88	13.31	up
POS_q232	Indole-3-methanamine	Others	10.07	81908.59	12.99	up
NEG_q67	Agnuside	Terpenoids	10.07	42403.47	12.04	up
NEG_q248	Narirutin	Flavonoids	10.07	38148.74	11.89	up
NEG_q144	D-(+)-Phenyllactic acid	Organic Acid	10.07	32642.23	11.66	up
POS_q230	Hyoscyamine sulfate hydrate	Alkaloids	10.07	11302.51	10.13	up
POS_q345	Phendimetrazine	Others	80997.95	10.07	-12.97	down
NEG_q3	1-(2,3,4-Trihydroxyphenyl)ethanone	Ketones, aldehydes, esters	85360.83	10.07	-13.05	down
POS_q74	(+)-Afzelechin	Flavonoids	90173.62	10.07	-13.13	down
NEG_q349	α-Linolenic acid	Lipid	161629.09	10.07	-13.97	down
NEG_q277	Piceatannol 3’-O-glucoside	Polyphenols	165019.46	10.07	-14.00	down
POS_q354	Pilocarpine	Alkaloids	189994.70	10.07	-14.20	down
POS_q160	D-Panthenol	Lipid	192657.53	10.07	-14.22	down
POS_q4	13,21-Dihydroeurycomanone	Terpenoids	327455.04	10.07	-14.99	down
POS_q239	Isovitexin 2’’-O-arabinoside	Flavonoids	1142648.16	10.07	-16.79	down
NEG_q294	Rhapontigenin 3’-O-glucoside	Polyphenols	1529583.11	10.07	-17.21	down

### 2.7 Conjoint analysis of DEGs and SCMs

**Figure 6:**
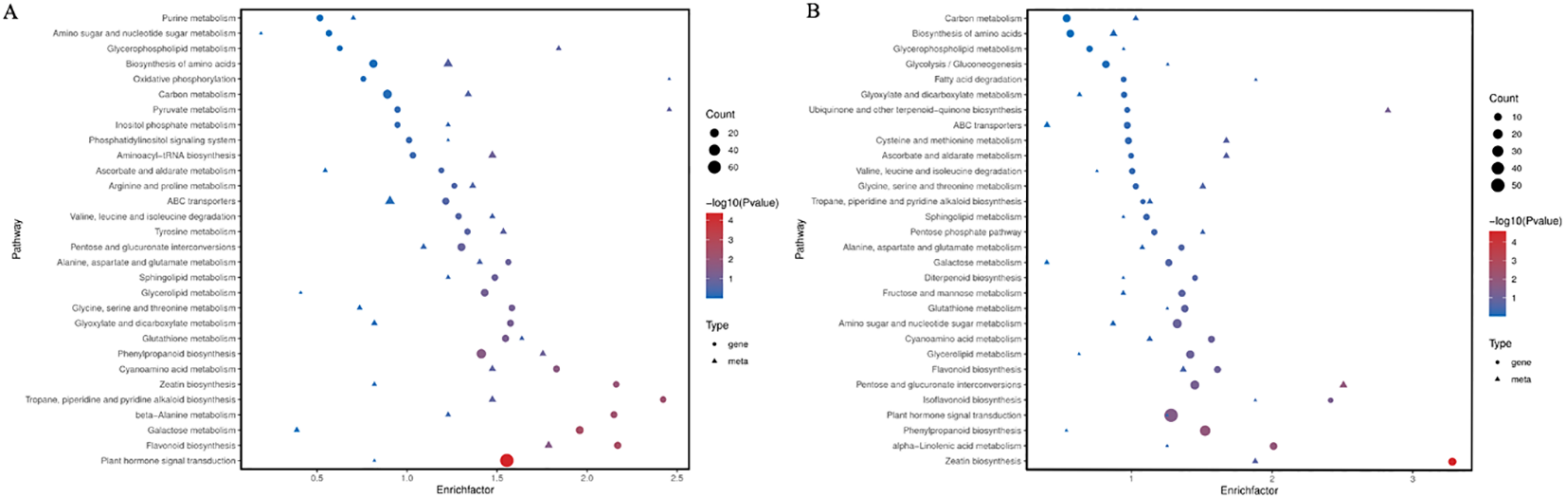
KEGG enrichment bubble plot of differentially expressed genes/metabolites. **(A)** BL-W. **(B)** BL-B.

In this project, two-way orthogonal partial least squares (O2PLS) was used to conduct an integrated analysis of the transcriptome and metabolome. The results revealed a significant correlation between the two datasets for the BP-W comparison and BP-B comparison (**Supplementary Figure S1**). The results of the KEGG enrichment bubble plot analysis of the DEGs/metabolites revealed that the top 5 pathways among the DEGs associated with the BP-W comparison were biosynthesis of tropane, piperidine, and pyridine alkaloids; flavonoid metabolism; zeaxanthin metabolism; β-alanine metabolism; and galactose metabolism. The top 5 pathways among the differential metabolites associated with the BP-W combination were pyruvate metabolism, oxidative phosphorylation, amino acid metabolism, phenylpropanoid metabolism, and flavonoid metabolism. The top 5 pathways among DEGs in the BP-B combination were related to zeaxanthin metabolism, alpha linolenic acid metabolism, isoflavone biosynthesis, flavonoid metabolism, and phenylpropanoid metabolism. The top 5 pathways among the DAMs in the BP-B comparison were related to the biosynthesis of ubiquinone and other terpenoid quinones, the interconversion of pentose and glucuronic acid, zeaxanthin metabolism, fatty acid metabolism, and isoflavone biosynthesis (**Figure 6**).

**Figure 7:**
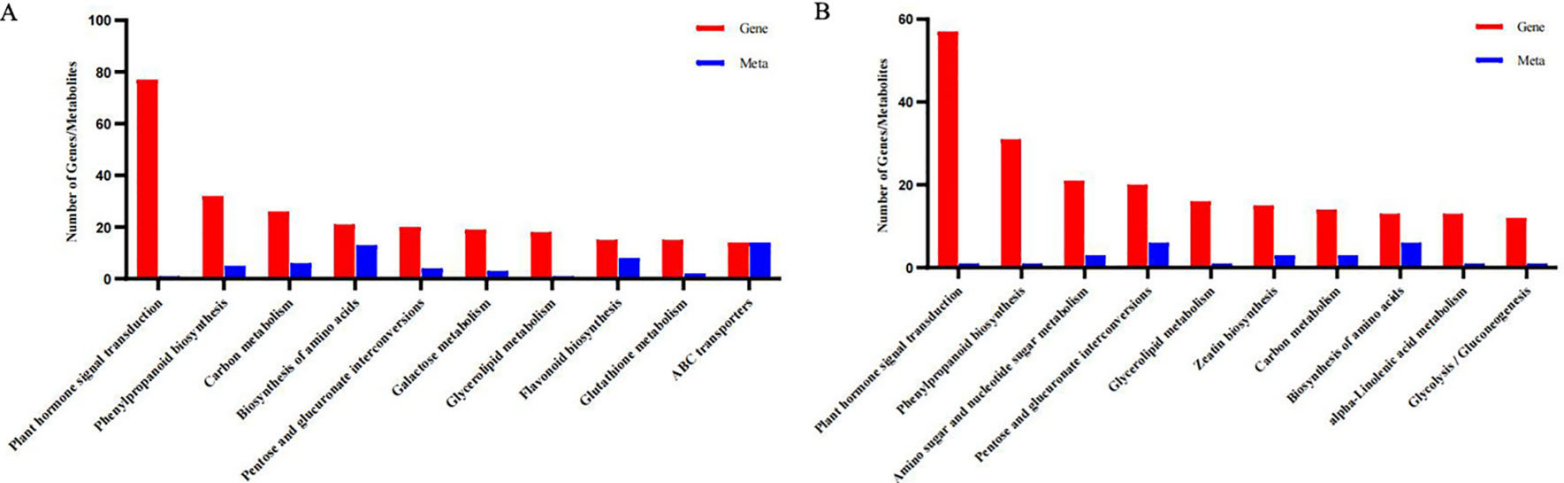
The top 10 pathways with the greatest numbers of genes/metabolites. **(A)** BL-W. **(B)** BL-B.

The top 10 KEGG pathways with the greatest numbers of co-related genes and metabolites identified in this project were visualized and analyzed (**Figure 7**), and the results revealed that the BP-W combination included plant hormone signal transduction, phenylpropanoid biosynthesis, carbon metabolism, biosynthesis of amino acids, pentose and glucuronate interconversions, galactose metabolism, glycerolipid metabolism, flavonoid biosynthesis, glutathione metabolism, and ABC transporter. The BP-B combination sequentially included plant hormone signal transduction, phenylpropanoid biosynthesis, amino sugar and nucleotide sugar metabolism, pentose and glucuronate interconversions, glycerolipid metabolism, zeatin biosynthesis, carbon metabolism, biosynthesis of amino acids, alpha-linolenic acid metabolism, and glycolysis/gluconeogenesis. Therefore, polarized light may be significantly associated with these metabolic pathways.

### 2.8 Analysis of DEGs and SCMs related to metabolism

The results of the DEG and SCM analyses above indicate that plant hormone signal transduction, zeatin biosynthesis, phenylpropanoid biosynthesis and flavonoid biosynthesis significantly differ between plants exposed to polarized light and ordinary light.

**Figure 8:**
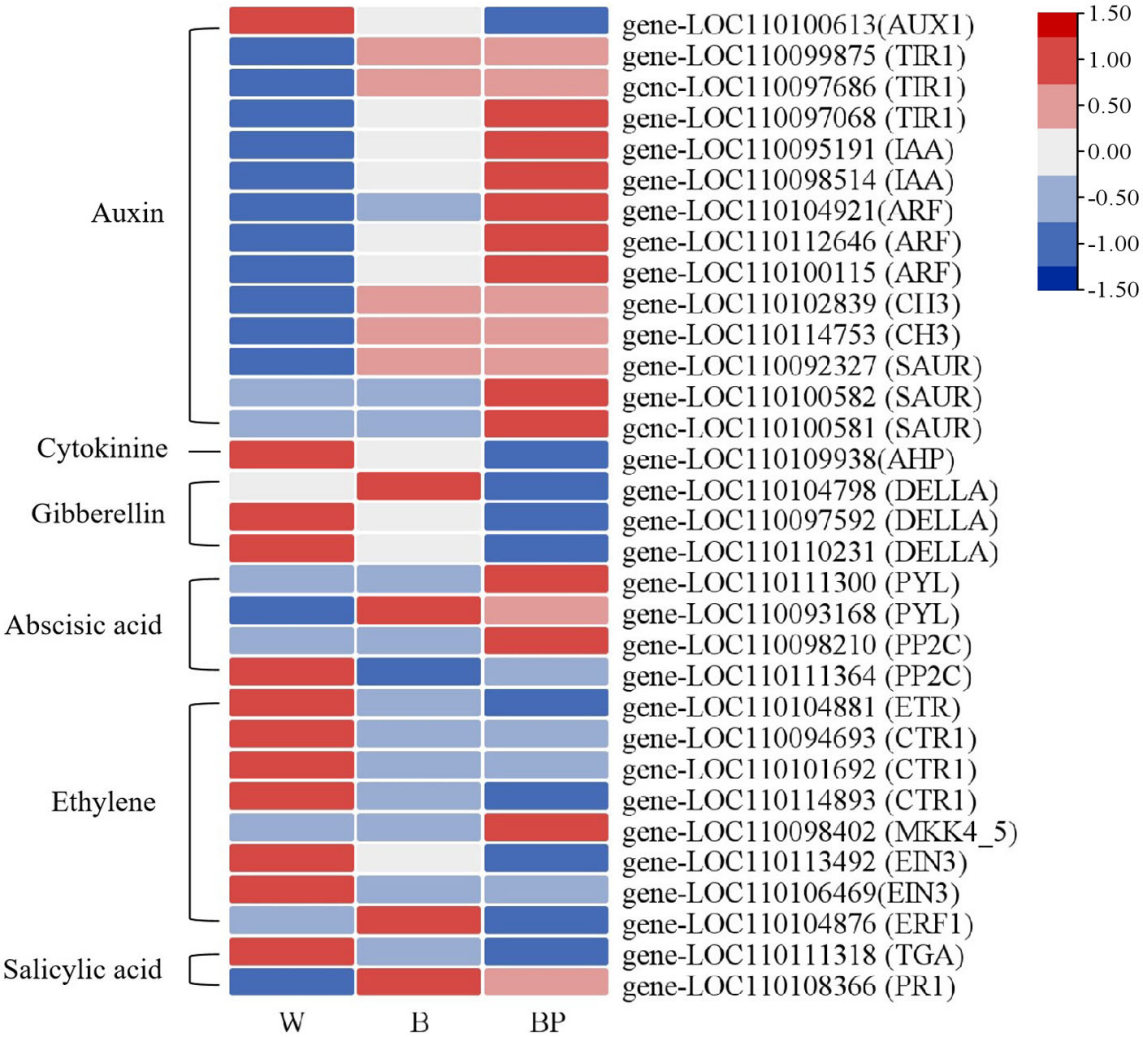
Cluster heatmap of differentially expressed genes involved in plant hormone signaling pathways.

Analysis of DEGs related to plant hormone signal transduction (**Figure 8**) revealed that the expression levels of 13 genes involved in auxin signal transduction, including *TIR1*, *IAA*, *ARF*, *CH3*, and *SAUR*, were greatest in the BP treatment, followed by those in the B treatment, and lowest in the W treatment. Only *AUX1* exhibited the opposite trend. The expression level of *AHP* in the context of cytokinin signal transduction was highest in treatment W, followed by treatment B, and lowest in treatment BP. The expression levels of the three *DELLA* genes involved in gibberellin signal transduction were highest in treatment W, followed by treatment B, and lowest in treatment BP. The expression level of *PYL* in abscisic acid signaling transduction was greatest in the BP treatment group. The expression levels of *ETR*, *CTR*1, and *EIN*3 involved in ethylene signaling transduction were greatest in the W treatment group, whereas the expression level of *ERF*1 was greatest in the B treatment group, and the expression level of *MKK*4_5*d* was greatest in the BP treatment group. The expression level of *TCA* in salicylic acid signaling transduction was greatest in the W treatment, and the expression level of *PR1* was greatest in the B treatment. Therefore, there were significant differences between polarized light and ordinary light in terms of plant hormone signaling pathways.

**Figure 9:**
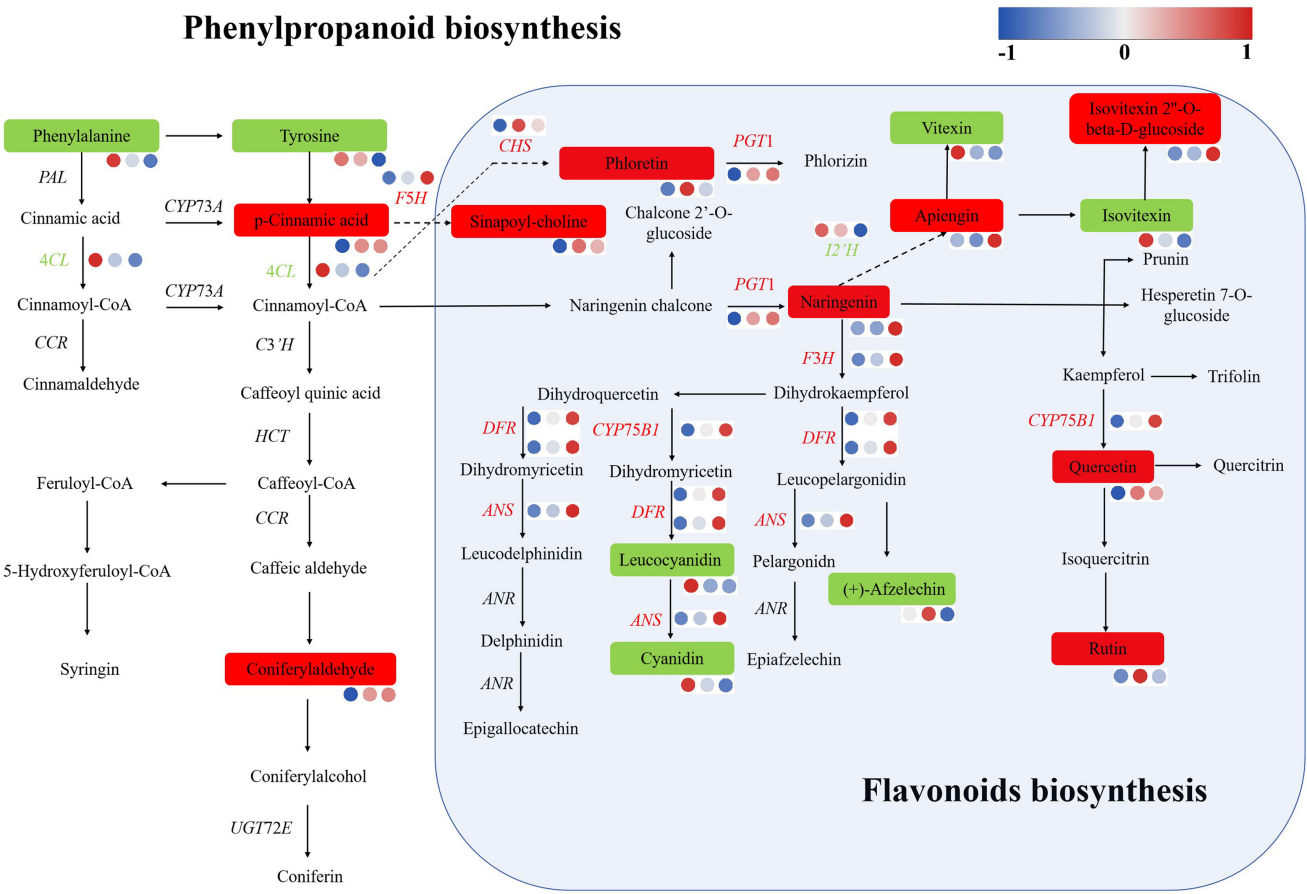
DEGs and SCMs involved in phenylpropanoid biosynthesis and flavonoid biosynthesis.

Analysis of DEGs and SCMs through phenylpropanoid biosynthesis and flavonoid biosynthesis identified upregulated DEGs including *F*5*H*, *CHS*, *F*3*H*, *CYP75B1*, *DFR*, *PGT1*, *ANS*, and *CYP81E* and downregulated DEGs including *4CL* and *I*2’*H*. The upregulated SCMs included apiengin, isovitexin 2’’-o-beta-d-glucoside, quercetin, rutin, phloretin, naringin, coumaric acid, sinapoyl-choline, and coniferaldehyde. The downregulated SCMs included phenylalanine, tyrosine, vitexin, isovitexin, leucocyanidin cyanidin, and (+)-afzelechin. Therefore, there were significant differences in the biosynthesis of phenylpropanoids and flavonoids between polarized light and ordinary light conditions (**Figure 9**).

### 2.9 qPCR identification of DEGs in *D. officinale* under different light treatments

**Figure 10:**
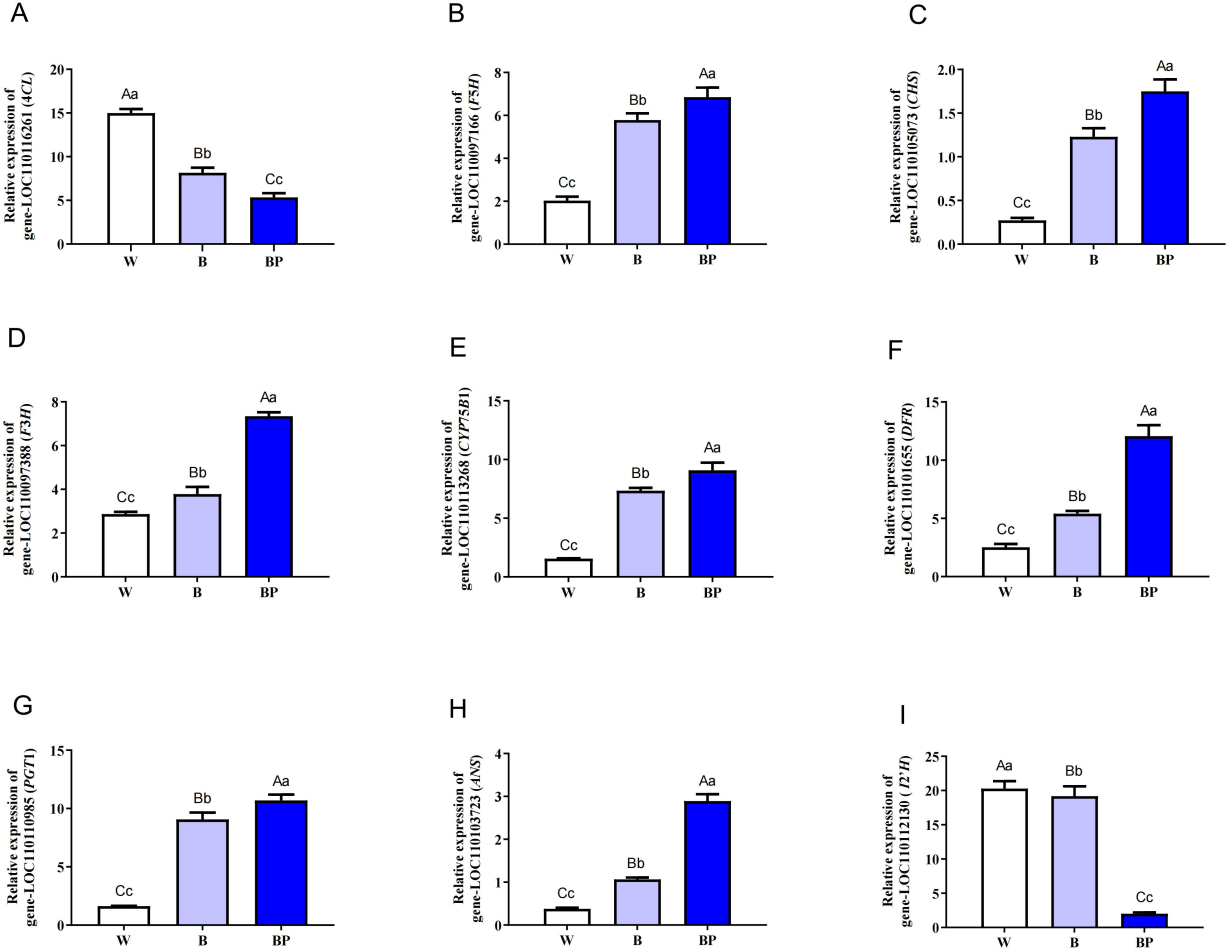
qPCR verification of DEGs in *D. officinale* under different light treatments. Different upper/lowercase letters indicate statistically significant differences at the 0.01/0.05 level, as determined by one-way ANOVA and Duncan’s test. **(A)** 4*CL* (4-coumarate--CoA ligase); **(B)** *F5H* (ferulate-5-hydroxylase); **(C)** *CHS* (chalcone synthase); **(D)** *F3H* (naringenin 3-dioxygenase); **(E)** *CYP75B1* (flavonoid 3’-monooxygenase); **(F)** *DFR* (bifunctional dihydroflavonol 4-reductase); **(G)** *PGT1* (phlorizin synthase); **(H)** *ANS* (anthocyanidin synthase); **(I)** *I2’H* (isoflavone/4’-methoxyisoflavone 2’-hydroxylase).

We conducted qPCR validation for 9 groups of mRNAs, and the results are shown in **Figure 10** (**Supplementary Tables S6**, **S7**). Genes related to phenylpropanoid biosynthesis and flavonoid biosynthesis play important roles in regulating the functional metabolites of *D. officinale* under polarized and ordinary light sources. For example, the expression levels of *F5H*, *CHS*, *F3H*, *CYP75B1*, *DFR*, *PGT1*, and *ANS* were highest in the BP treatment group, followed by those in the B treatment group, and lowest in the W treatment group. However, *4CL* and *I2’H* exhibited the exact opposite pattern, with the highest expression levels in the W treatment group, followed by the B treatment group, and lowest in the BP treatment group. Therefore, polarized light may affect the accumulation of functional metabolites in *D. officinale* through the positive regulation of *F5H*, *CHS*, *F3H*, *CYP75B1*, *DFR*, *PGT1*, and *ANS* expression, whereas the expression of *4CL* and *I*2’*H* are negatively regulated.

## 3 Discussion

### 3.1 Significant differences in plant hormone signal transduction were observed in response to blue polarized light and ordinary light sources in *D. officinale*

The results of the KEGG analysis of the DEGs revealed that plant hormone signal transduction and zeatin biosynthesis were enriched among the top 20 DEGs (**Figure 4**). A KEGG enrichment bubble plot analysis of the differentially expressed genes/metabolites revealed that zeatin biosynthesis was among the top 5 enriched pathways (**Figure 6**). Analysis of the top 10 pathways with the greatest number of DEGs/DAMs revealed that plant hormone signal transduction ranked first (**Figure 7**). The DEG analysis in this study revealed significant differences in genes involved in the signaling pathways of auxin, gibberellin, abscisic acid, and ethylene (**Figure 8**). Therefore, there are significant differences in plant hormone signal transduction in response to polarized light sources and ordinary light sources in *D. officinale*. This may be due to the specific regulation of hormone pathways by light signals and the combined driving force of the physical properties of polarized light.

Polarized light can increase the efficiency of hormone signal transduction. Due to its directional consistency, polarized light can quickly activate photosensitive pigments (such as phyB) and blue light receptors, significantly accelerating the accumulation of calcium ion flow and ROS signals (such as H_2_O_2_) (Hong et al., 2025). These signaling molecules directly regulate the activity of hormone-responsive factors such as auxin (Aux/IAA) and ethylene (ERF), among which ROS can bypass the classical Aux/IAA protein degradation pathway and drive transcription reactions through secondary messengers such as cyclic adenosine monophosphate (Chen H. et al., 2025). However, it is difficult for scattered light from ordinary light sources to trigger ROS bursts of equal intensity, resulting in lower efficiency of hormone signal transduction. In addition, in *D. officinale*, the ethylene-responsive factor DoERF5 negatively regulates the key gene DoWOX4 for polysaccharide synthesis. Polarized light enhances the blue light component and inhibits the expression of DoERF5, thereby alleviating its inhibitory effect on DoWOX4 and promoting mannan synthesis (Danqi et al., 2025),. Under normal light conditions, the inhibitory effect of DoERF5 is more significant, leading to a weakened metabolic response.

The key function of chloroplast reverse signaling is to counteract photooxidative damage. Polarized light has strong penetrating power, greatly improving photosynthetic efficiency, but it also causes excessive accumulation of chloroplast precursor proteins (cpPOSs), inducing a surge in chloroplast reactive oxygen species (cpROS), which promotes the expression of cytoplasmic molecular chaperones (such as ClpB1/HOT1) and heat shock proteins (HSPs) through reverse signaling (Su and Gassmann, 2023). cpROS interact with auxin and ethylene signaling, preferentially initiating the flavonoid synthesis pathway to combat photooxidative damage (Su and Gassmann, 2023). Ordinary light sources have low light energy utilization efficiency and thus induce less accumulation of precursor proteins, making them less likely to activate the same level of cpROS in the HSP defense axis.

*D. officinale* has special metabolic adaptability. *D. officinale* continuously absorbs CO_2_ in the dark and converts it into acidic intermediates, providing a rich carbon skeleton for hormone-regulated metabolism under light conditions. Polarized light further enhances this effect through efficient photosynthesis, promoting the synthesis of secondary metabolites such as flavonoids. As an epiphytic orchid, *D. officinale* has evolved a high sensitivity to polarized blue light from the forest canopy. Its ERF5–WOX4 regulatory module and ROS-mediated hormone pathway are more susceptible to polarized light activation, thereby optimizing its photoprotective mechanism (Chen L. et al., 2025).

In summary, polarized light enhances the efficiency of light receptor activation through its physical properties, synergizes with nonclassical transduction pathways (such as cyclic nucleotide pathways), and drives reverse signaling cascades in chloroplasts, ultimately leading to the directional differentiation of metabolic responses in *D. officinale*.

### 3.2 Phenylpropanoid metabolism and flavonoid metabolism significantly differed in response to blue-polarized light and ordinary light sources in *D. officinale*

In this study, GO analysis revealed that the DEGs were significantly enriched in phenylpropanoid metabolism (**Figure 3**). KEGG analysis of the DEGs revealed that flavonoid biosynthesis, isoflavonoid biosynthesis, and flavone and flavonol biosynthesis were enriched in the top 20 DEGs (**Figure 4**). A KEGG enrichment bubble plot analysis of the DEGs/DAMs revealed that flavonoid metabolism and phenylpropanoid metabolism were among the top 5 most enriched pathways (**Figure 6**). Analysis of the top 10 pathways with the greatest number of DEGs/DAMs revealed that phenylpropanoid biosynthesis ranked second, and flavonoid biosynthesis ranked eighth (**Figure 7**). The combined analysis of DEGs and SCMs in this study revealed many DEGs and metabolic pathways involved in phenylpropanoid biosynthesis and flavonoid biosynthesis (**Figure 8**).

The differences in phenylpropanoid metabolism and flavonoid metabolism in *D. officinale* in response to polarized light sources and ordinary light sources are due mainly to the differential regulation of key enzymes in metabolic pathways by light signals, as well as the impact of the unique physical properties of polarized light on the efficiency of light receptor activation, as follows: (1) The enhancement effect of polarized light on optical signal transmission. Polarized light has directionally consistent electromagnetic oscillation characteristics, which can more efficiently activate photosensitive pigments (such as phyB) and phototropin in plant cells, significantly improving the efficiency of light signal transduction. However, ordinary light sources (such as white light) contain chaotic polarization directions and have lower signal transmission efficiency. Efficient activation of photoreceptors leads to faster accumulation of calcium ion flow and ROS signaling molecules (such as H_2_O_2_), which in turn increase the expression of transcription factors (such as MYB and bHLH) through the MAPK cascade, preferentially initiating the flavonoid synthesis pathway (Zeidler, 2022; Pan et al., 2016; Hernández et al., 2009). (2) Differential regulation of metabolic pathways. The key upstream enzymes involved in flavonoid synthesis, phenylalanine ammonia lyase (PAL) and chalcone synthase (CHS), are strongly induced by blue/ultraviolet light bands (Pan et al., 2016; Hernández et al., 2009; Hu and Lv, 2015). Polarized light contains a high proportion of blue light components, significantly increasing CHS activity and promoting the synthesis of flavonols such as quercetin (Pan et al., 2016; Hernández et al., 2009; Hu and Lv, 2015). Flavonoids act as photoprotectors, accelerating their accumulation under polarized light to resist oxidative stress caused by directional light radiation (Wang et al., 2020). The contents of antioxidant substances such as quercetin derivatives in *D. officinale* increase under polarized light. The initiator enzyme PAL of the phenylpropanoid pathway is regulated by light but is less affected by light quality. Its products (such as lignin and coumarin) are mainly structural support substances, and there is little difference in accumulation under different types of light sources (polarized vs. ordinary) (Cho et al., 2024). (3) The biological advantages of polarized light. Polarized light can affect the leaf epidermis directly to mesophyll cells, improving photosynthetic efficiency and providing more carbon skeletons (such as phenylalanine) for secondary metabolism. Flavonoids accumulate directionally in cell walls and vacuoles under polarized light, forming a localized high-concentration protective layer that effectively quenches reactive oxygen species (such as singlet oxygen) generated by directional light radiation, whereas scattered light from ordinary light sources does not easily trigger such targeted defenses (Wang et al., 2020). (4) Evolutionary factors of environmental adaptability. As an epiphytic orchid, *D. officinale* has long adapted to the polarized light environment under the forest canopy (such as the polarized blue light reflected by leaves). Its flavonoid metabolism pathway has evolved a high sensitivity to polarized light to optimize light protection strategies. This adaptability is weakly expressed in the random light field of ordinary light sources (Jia et al., 2021).

In summary, polarized light activates blue light receptors efficiently, enhances ROS signaling, and provides directional light stress, with a triple mechanism prioritizing flavonoid metabolism; however, the metabolism of phenylpropanoids has low sensitivity to functional requirements and light regulation, and the response difference is not significant.

### 3.3 Microtubule activity, UDP-glycosyltransferase activity and microtubule binding significantly differ in *D. officinale* exposed to blue polarized light relative to ordinary light

In this study, cellular component analysis of the DEGs via GO revealed that the P value of the microtubules term was less than 0.01 for the BP-W and BP-B comparisons. GO molecular function analysis of the DEGs revealed that the P values of the UDP glycosyltransferase activity and microtubule binding terms were less than 0.01 for the BP-W and BP-B comparisons. Therefore, in the response of *D. officinale* to blue polarized light and ordinary light, there are significant differences in the pathways associated with microtubules, UDP glycosyltransferase activity, and microtubule binding.

With respect to the microtubule pathway, blue polarized light, as a special optical signal, may be recognized by specific photoreceptors in *D. officinale* cells because of its polar characteristics, triggering signal transduction pathways different from those triggered by ordinary light. The microtubule system inside plant cells is sensitive to light signals. Some studies have shown that blue light can redirect microtubules in the plant cell cortex, changing their arrangement from perpendicular to the cell growth axis to longitudinal, to regulate cell elongation and growth (Zhang et al., 2024). Blue polarized light may increase the specificity of the degree, direction, and related dynamic processes of microtubule rearrangement, which differs from the changes in microtubules caused by ordinary blue light. This may be because the direction of the polarized light provides additional spatial orientation information for cells, prompting them to adjust their physiological responses through the microtubule system (Chukhutsina et al., 2020).

The difference in UDP glycosyltransferase pathway activity may be due to the differential regulation of metabolism and physiological status in *D. officinale* by blue polarized light and ordinary light. UDP glycosyltransferase mainly participates in the catalysis of glycosyl transfer reactions and can modify hormones, secondary metabolites, etc., in plants, affecting the activity and function of substances (Yang et al., 2022; Bielczynski et al., 2016). Blue polarized light may alter the activity and expression levels of transcription factors in cells through unique light signal transduction, thereby regulating the transcription of UDP glycosyltransferase genes and causing changes in enzyme activity. Compared with normal light conditions, these conditions can lead to differences in the degree of glycosylation of substrates and the selection of modification sites, ultimately affecting the adaptation of plants to light environments and their own growth and development processes (Huang et al., 2018; Fristedt et al., 2015; Anja et al., 2016; Ren et al., 2015).

With respect to the microtubule binding pathway, the changes in the intracellular signal induced by blue polarized light have different regulatory effects on the function and activity status of microtubule binding proteins. Microtubule binding proteins can specifically bind to microtubules and play important regulatory roles in microtubule assembly, stability, and dynamic changes. Compared with ordinary light, blue polarized light may activate signaling pathways such as phosphorylation cascades within cells, resulting in differences in the phosphorylation and modification states of microtubule-binding proteins. This can alter their affinity, binding mode, and binding sites with microtubules. Ultimately, this leads to significant differences in physiological processes mediated by microtubule binding pathways, such as organelle transport and cell morphology maintenance, in *D. officinale* under blue polarized light relative to that under ordinary light (Kyaw et al., 2023; Lima et al., 2018).

## 4 Materials and methods

### 4.1 Plant materials and light treatment

The tissue culture-generated plants used in this project were all *D. officinale* with consistent growth, with a seedling height of approximately 2 cm, a leaf width of approximately 2–3 mm, and 3–4 true leaves. The *D. officinale* tissue culture-generated seedlings were placed in a light incubator for 60 days under the same conditions: either white light (W), blue light (B, 450 nm), or blue polarized light (BP, 450 nm, 90°); light intensity (200/μmol·m^-2^·S^-2^); light exposure time (12 h/d); humidity (55% to 60%); and temperature (25 ± 2 °C). The medium consisted of 1/2 MS + 6 g/L agar+1 g/L activated carbon+30.0 g/L sucrose (pH 5.8). W served as the control group, while B and BP were the experimental groups. Comparisons of samples treated with BP versus W, BP versus B and B versus W were named BP-W, BP-B, and B-W, respectively. The control group and experimental group samples were rapidly frozen in liquid nitrogen and stored in a -80 °C freezer for future use.

### 4.2 RNA extraction and sequencing

In this study, plants exposed to three treatments, W, B, and BP, were subjected to high-throughput sequencing, with three biological replicates for each treatment. RNA quantification and qualification were conducted following the methods described by Li et al. (Li et al., 2021). Subsequently, library preparation and high-throughput sequencing were carried out using the Illumina HiSeq platform (Beijing, China). All sequencing data for *D. officinale* subjected to different light treatments were deposited in the National Center for Biotechnology Information (NCBI) Sequence Read Archive (accession number PRJNA045164 and PRJNA045169).

Adaptor sequences and low-quality sequence reads were eliminated from the datasets. After data processing, the raw sequences were converted into clean reads. These clean reads were subsequently mapped to the reference genome sequence (*D. officinale* v1.0) (Zhang et al., 2016). Only reads with zero or one mismatch were further analyzed and annotated using the reference genome. HISAT2 tool software was employed for mapping with the reference genome.

### 4.3 Analysis of differentially expressed genes

The input data for differential gene expression analysis were obtained from the read count data obtained from gene expression level analysis. DESeq2 software was used for the analysis. The main process was as follows: (1) read count standardization; (2) calculation of the hypothesis test probability; and (3) multiple hypothesis testing correction to obtain the false discovery rate (FDR). Genes with a P value<0.01 and log2(fold change)>1 in the above analysis were selected as differentially expressed genes (Love et al., 2014).

### 4.4 Functional analysis of differentially expressed genes

A functional analysis of all genes that exhibited significant differences was performed with the GO and KEGG databases used as references, on the basis of the principle of hypergeometric distribution, and functional enrichment and pathway enrichment operations were implemented using ClusterProfiler software (Yu et al., 2012).

### 4.5 Detection of relative expression levels of miRNAs and mRNAs

The Roche LightCycler 480 platform was used to detect relative mRNA expression levels. Using the FastKing gDNA Dispelling RT SuperMix kit provided by Tiangen Biochemical Technology Co., Ltd. (Beijing, China), mRNA was reverse transcribed with actin as the reference gene for mRNA (Shen et al., 2017). A TB Green Premix Ex Taq II kit (Dalian, China) was used to detect the obtained cDNA on a fluorescence quantitative PCR instrument, and the relative expression level was calculated according to the 2^-ΔΔCt^ method. Each group was prepared with 3 biological replicates, and 3 technical replicates were prepared for each reaction. The details of the primers used in the qRT–PCR experiments are shown in **Supplementary Table S8**.

### 4.6 Wide targeted metabolome detection

#### 4.6.1 Metabolite extraction

The sample extracts were analyzed using a UPLC–ESI–MS/MS system (UPLC: Waters Acquity I-Class PLUS; MS: Applied Biosystems 6500+ Q TRAP) (Wang et al., 2016). The analytical conditions were as follows. For UPLC, a Waters HSS-T3 column (1.8 µm, 2.1 mm * 100 mm) was used. The mobile phase consisted of solvent A (pure water with 0.1% formic acid) and solvent B (acetonitrile with 0.1% formic acid and 5 mM ammonium acetate). The sample measurements used a gradient program starting at 98% A and 2% B for 1.5 min. Then, the gradient changed linearly to 50% A and 50% B over 3.5 min and then to 2% A and 98% B over 4 min and held for 1 min. Subsequently, the gradient was adjusted to 98% A and 2% B over 1 min and held for 3 min. The flow velocity was 0.35 mL/min, the column oven was set to 50 °C, and the injection volume was 2 µL. The effluent was connected to an ESI-triple quadrupole-linear ion trap (QTRAP)-MS.

#### 4.6.2 LC–MS/MS analysis

The ESI source operation parameters were as follows: source temperature, 550 °C; ion spray voltage (IS), 5500 V (positive ion mode)/-4500 V (negative ion mode); ion source gas I (GSI); gas II (GSII); and curtain gas (CUR), which were set at 50, 55, and 35 psi, respectively; and collision-activated dissociation (CAD) was set to medium. Instrument tuning and mass calibration were performed with 10 and 100 μmol/L polypropylene glycol solutions in QQQ and LIT modes, respectively. QQQ scans were acquired as MRM experiments with the collision gas (nitrogen) set to medium. DP and CE for individual MRM transitions were optimized further. A specific set of MRM transitions was monitored for each period according to the metabolites eluted within this period (Garcia and Barbas, 2011).

#### 4.6.3 Data analysis

After the original peak area information was normalized to the total peak area, follow-up analysis was conducted. Principal component and Spearman correlation analyses were used to assess the repeatability of the within-group samples and quality control samples. The identified compounds were searched for classification and pathway information in the KEGG (Kanehisa and Goto, 2000), HMDB (Wishart et al., 2018) and lipidmaps databases. Afterward, according to the grouping information, the difference multiples were calculated and compared, and the t test was used to calculate the difference significance p value of each compound. The R language package ropls was used for OPLS-DA modeling, and 200 permutation tests were performed to verify the reliability of the model. The VIP value of the model was calculated via multiple cross-validation. Differentially abundant metabolites were screened by combining the difference multiple, P value and VIP value of the OPLS-DA model (Thévenot et al., 2015), with screening criteria of FC>1, P value<0.05 and VIP>1. Finally, the differential enrichment of metabolites in the KEGG pathway was calculated using the hypergeometric distribution test.

### 4.7 Data analysis

At least 3 biological replicates were evaluated for the gene expression and functional metabolite data analyses. Duncan’s one-way analysis of variance (ANOVA) was implemented in SPSS V19.0 to analyze the different indicators. GraphPad Prism 6.0 software and OmicShare online software were used for mapping.

## Data Availability

The datasets presented in this study can be found in online repositories. The names of the repository/repositories and accession number(s) can be found in the article/**Supplementary Material**.

## References

[B1] Agrawal N. (2025). Manipulating floquet-driven topological insulator with off-resonant elliptically polarized light in presence of hexagonal Fermi surface warping. J. Phys. Condens Matter 37, 085301. doi: 10.1088/1361-648X/ad98db, PMID: 39612573

[B2] Anja S. Iris S. Andrei H. Chiara G. Marion E. (2016). The evolutionarily conserved protein PHOTOSYNTHESIS AFFECTED MUTANT71 in requiring efficient manganese uptake at the thylakoid membrane in *Arabidopsis*. Plant Cell 28, 892–910. doi: 10.1105/tpc.15.00812, PMID: 27020959 PMC4863382

[B3] Bielczynski L. W. Gert S. Roberta C. (2016). Effect of light acclimation on the organization of photosystem II super- and sub-complexes in *Arabidopsis* thaliana. Front. Plant Sci. 7, 105. doi: 10.3389/fpls.2016.00105, PMID: 26925068 PMC4756287

[B4] Chen H. Qi L. Zou M. Lu M. Kwiatkowski M. Pei Y. . (2025). TIR1-produced cAMP as a second messenger in transcriptional auxin signalling. Acta Pharmacol. Sin. 640, 1011–1016. doi: 10.1038/s41586-025-08669-w, PMID: 40044868 PMC12018254

[B5] Chen L. Duan S. Huang J. Hu L. Liu S. Lan Q. . (2025). Integrated metabolomic and transcriptomic analysis in revealing variation in the metabolites of *Dendrobium officinale*, *Dendrobium huoshanense*, *Dendrobium nobile*. Phytochem. Anal. 36, 36–47. doi: 10.1002/pca.3429, PMID: 39118423

[B6] Chen X. Chen C. Ma C. Zhao L. Liu H. Chen Y. (2025). *Dendrobium officinale* polysaccharide in attenuating type 2 diabetes in mice model by modulating gut microbiota and alleviating intestinal mucosal barrier damage. Food Sci. Hum. Wellness 14, 112–123. doi: 10.26599/FSHW.2024.9250007

[B7] Cho J. S. Kim M. H. Jang H. A. Choi H. Jeon H. W. Lee H. . (2024). Functional impacts of *PtrMYB*203 on phenylpropanoid pathway regulation and wood properties in hybrid poplar. Plant Physiol. Biochem. 216, 109118. doi: 10.1016/j.plaphy.2024.109118, PMID: 39270565

[B8] Chukhutsina V. U. Liu X. Xu P. Croce R. (2020). Light-harvesting complex II as an antenna of photosystem I in dark-adapted plants. Nat. Plants 7, 1–9. doi: 10.1038/s41477-020-0693-4, PMID: 32572215

[B9] Danqi Z. Hongyu S. Jing C. Si C. Duan J. Liu Z. J. . (2025). The ERF5-WOX4 transcription factor regulatory module in controlling mannan biosynthesis in the orchid *Dendrobium officinale*. Plant Physiol. 198, kiaf273. doi: 10.1093/plphys/kiaf273, PMID: 40561509

[B10] Favero D. S. (2020). A chloroplast-derived signal in attenuating growth in red light by acting on the phyB-PIF pathway. Plant Physiol. 183, 1408–1409. doi: 10.1104/pp.20.00819, PMID: 32747485 PMC7401120

[B11] Fristedt R. Herdean A. Blaby-Haas C. E. Fristedt R. L. (2015). PHOTOSYSTEM II PROTEIN33, a protein conserved in the plastid lineage, in association with the chloroplast thylakoid membrane and providing stability to photosystem II supercomplexes in *Arabidopsis*. Plant Physiol. 167, 481–492. doi: 10.1104/pp.114.253336, PMID: 25511433 PMC4326745

[B12] Garcia A. Barbas C. (2011). Gas chromatography-mass spectrometry (GC-MS)-based metabolomics. Methods Mol. Biol. 2, 191–204. doi: 10.1007/978-1-61737-985-7_11, PMID: 21207291

[B13] Hernández I. Alegre L. Van Breusegem F. Munné-Bosch S. (2009). Flavonoids as antioxidants in plants. Trends Plant Sci. 14, 125–132. doi: 10.1016/j.plantsci.2012.07.014, PMID: 19230744

[B14] Hong Z. H. Zhu L. Gao L. L. Zhu Z. Su T. Krall L. . (2025). Chloroplast precursor protein preClpD overaccumulation in triggering multilevel reprogramming of gene expression and a heat shock-like response. Nat. Commun. 16, 3777. doi: 10.1038/s41467-025-59043-3, PMID: 40263324 PMC12015282

[B15] Hu H. T. Lv X. M. (2015). Production of plant flavonoids in microbial cell factories: key enzyme mining and heterologous biosynthesis. Food Sci. 46, 1–17. doi: 10.7506/spkx1002-6630-20250118-139

[B16] Huang W. Yang Y. J. Zhang S. B. Tao L. (2018). Cyclic electron flow around photosystem I in promoting ATP synthesis possibly helping the rapid repair of photodamaged photosystem II at low light. Front. Plant Sci. 9, 239. doi: 10.3389/fpls.2018.00239, PMID: 29535751 PMC5834426

[B17] Jia N. Wang J. J. Liu J. Jiang J. Fan B. (2021). DcTT8, a bHLH transcription factor, in regulating anthocyanin biosynthesis in *Dendrobium candidum*. Plant Physiol. Biochem. 162, 603–612. doi: 10.1016/j.plaphy.2021.03.006, PMID: 33774465

[B18] Kanehisa M. Goto S. (2000). KEGG: kyoto encyclopedia of genes and genomes. Nucleic Acids Res. 28, 27–30. doi: 10.1093/nar/28.1.27, PMID: 10592173 PMC102409

[B19] Kulchin Y. N. Kozhanov S. O. Kholin A. S. Subbotin E. P. Subbotina N. I. Gomolsky A. S. . (2024). Linearly polarized light in the dynamics of maize plants development. Bull. Russ Acad. Sci. Phys. 88, S357–S360. doi: 10.1109/iclo59702.2024.10624190

[B20] Kyaw A. Roepke K. Arthur T. H. K. P. (2023). Conformation of influenza AM2 membrane protein in nanodiscs and liposomes. Biochim. Biophys. Acta 1865, 184152. doi: 10.1016/j.bbamem.2023.184152, PMID: 36948480 PMC10175228

[B21] Li H. S. Ye W. Gang S. Chen X. H. Fang Y. Sun G. (2021). RNA sequencing-based exploration in the effects of far-red light on lncRNAs involved in the shade-avoidance response of D. officinale. PeerJ 9, e10769. doi: 10.7717/peerj.10769, PMID: 33614278 PMC7883695

[B22] Lima G. M. Teixeira P. C. N. Cláudia M. L. L. T. Filócomo D. Lage C. L. S. (2018). Influence of spectral light quality on the pigment concentrations and biomass productivity of *Arthrospira platensis*. Algal Res. 31, 157–166. doi: 10.1016/j.algal.2018.02.012

[B23] Love M. I. Huber W. Anders S. (2014). Moderated estimation of fold change and dispersion for RNAseq data with DESeq2. Genome Biol. 15, 550–558. doi: 10.1186/s13059-014-0550-8, PMID: 25516281 PMC4302049

[B24] Meng X. L. Zhao X. Ding X. Li Y. Cao G. D. (2020). Integrated functional omics analysis in flavonoid related metabolism in *AtMYB*12 transcript factor overexpressed tomato. J. Agric. Food Chem. 68, 6776–6787. doi: 10.1021/acs.jafc.0c01894, PMID: 32396374

[B25] Monte E. (2020). AHL transcription factors in inhibiting growth promoting PIFs in plant biology. Curr. Biol. 30, 354–356. doi: 10.1016/j.cub.2020.03.035, PMID: 32315635

[B26] Pan J. Q. Tong X. R. Guo B. L. (2016). Progress of effects of light on plant flavonoids. Chin. J. Chin. Mater Med. 41, 3897–3903. doi: 10.4268/cjcmm20162103, PMID: 28929672

[B27] Ren J. Cai B. Xiangwei H. E. Yao H. J. Du S. J. Shang F. N. (2015). The relationship between stomatal movement and light intensity gradient in three dendrobium species compared with typical CAM plants. Agric. Biotechnol. 4, 2164–4993. doi: CNKI:SUN:AGBT.0.2015-02-009

[B28] Shen C. Guo H. Chen H. Shi Y. J. Meng Y. J. Lu J. J. . (2017). Identification and analysis of genes associated with the synthesis of bioactive constituents in *Dendrobium officinale* using RNA-Seq. Sci. Rep. 7, 187–198. doi: 10.1038/s41598-017-00292-8, PMID: 28298629 PMC5412657

[B29] Su J. Gassmann W. (2023). Cytoplasmic regulation in chloroplast ROS accumulation during effector-triggered immunity. Front. Plant Sci. 14, 1127833. doi: 10.3389/fpls.2023.1127833, PMID: 36794218 PMC9922995

[B30] Thévenot E. A. Roux A. Xu Y. Ezan E. Junot C. (2015). Analysis of the human adult urinary metabolome variations with age, body mass index, and gender by implementing a comprehensive workflow for univariate and OPLS statistical analyses. J. Proteome Res. 14, 3322–3335. doi: 10.1021/acs.jproteome.5b00354, PMID: 26088811

[B31] Wang P. Chen S. Gu M. Chen X. Chen X. Yang J. . (2020). Exploration of the effects of different blue LED light intensities on flavonoid and lipid metabolism in tea plants via transcriptomics and metabolomics. Int. J. Mol. Sci. 21, 4606–4615. doi: 10.3390/ijms21134606, PMID: 32610479 PMC7369854

[B32] Wang J. Zhang T. Shen X. Liu J. Zhao D. L. Sun Y. W. (2016). Serum metabolomics in early diagnosis of esophageal squamous cell carcinoma by UHPLC-QTOF/MS. Metabolomics 12, 116–129. doi: 10.1007/s11306-016-1050-5

[B33] Wen W. Alseekh S. Fernie A. R. (2020). Conservation and diversification of flavonoid metabolism in the plant kingdom. Curr. Opin. Plant Biol. 55, 100–108. doi: 10.1016/j.pbi.2020.04.004, PMID: 32422532

[B34] Wishart D. S. Feunang Y. D. Marcu A. Guo A. C. Liang K. Rosa V. F. . (2018). HMDB 4.0: the human metabolome database for 2018. Nucleic Acids Res. 46, D608–D617. doi: 10.1093/nar/gkx1089, PMID: 29140435 PMC5753273

[B35] Wu Y. H. Chen X. G. Li N. Q. Li T. Q. Anbazhakan R. Gao J. Y. (2025). Core mycorrhizal fungi in promoting seedling growth in *Dendrobium officinale*: an important medicinal orchid. Plants 14, 2223–2247. doi: 10.3390/plants14071024, PMID: 40219092 PMC11990756

[B36] Wu R. Tian Z. M. Zheng G. H. (2020). Overexpression of CHS genes from Trifolium repens in increasing tobacco flavonoid content. Pratacult Sci. 37, 305–313. doi: 10.11829/j.issn.1001-0629.2019-0288

[B37] Yang Z. Liu B. Su J. Liao J. K. Lin C. T. (2017). Cryptochromes in orchestrating transcription regulation of diverse blue light responses in plants. Photochem. Photobiol. 93, 112–127. doi: 10.1111/php.12663, PMID: 27861972 PMC6167254

[B38] Yang Y. J. Shi Q. Sun H. Mei R. Q. Huang W. (2022). Differential response of the photosynthetic machinery to fluctuating light in mature and young leaves of *Dendrobium officinale*. Front. Plant Sci. 12, 829783. doi: 10.3389/fpls.2021.829783, PMID: 35185969 PMC8850366

[B39] Yang Y. J. Tan S. L. Sun H. Huang J. L. Huang W. Zhang S. B. (2021). Photosystem I in tolerance to fluctuating light under moderate heat stress in two orchids *Dendrobium officinale* and *Bletilla striata*. Plant Sci. 303, 110795. doi: 10.1016/j.plantsci.2020.110795, PMID: 33487367

[B40] Yu G. Wang L. G. Han Y. He Q. Y. (2012). ClusterProfiler: an R package in comparing biological themes among gene clusters. Omics 16, 284–287. doi: 10.1089/omi.2011.0118, PMID: 22455463 PMC3339379

[B41] Zeidler M. (2022). Physiological analysis of phototropic responses to blue and red light in *Arabidopsis*. Methods Mol. Biol. 2494, 37–45. doi: 10.1007/978-1-0716-2297-1_4, PMID: 35467199

[B42] Zeng B. Yan Y. Zhang Y. (2024). *Dendrobium officinale* polysaccharide (DOP) in inhibiting cell hyperproliferation, inflammation and oxidative stress to improve keratinocyte psoriasis-like state. Adv. Med. Sci. 69, 167–175. doi: 10.1016/j.advms.2024.03.005, PMID: 38521458

[B43] Zhang X. Abrahan C. Colquhoun T. A. Liu C. J. (2017). A proteolytic regulator in controlling chalcone synthase stability and flavonoid biosynthesis in *Arabidopsis*. Plant Cell 29, 1157–1174. doi: 10.1105/tpc.16.00855, PMID: 28446542 PMC5466025

[B44] Zhang Y. Z. Li K. Qin B. Y. Guo B. Y. Zhang J. P. Bao Q. B. (2024). Structure of cryptophyte photosystem II-light-harvesting antennae supercomplex. Nat. Commun. 15, 4999–5009. doi: 10.1038/s41467-024-49453-0, PMID: 38866834 PMC11169493

[B45] Zhang L. Li T. Su S. Zuo Z. (2021). COP1/SPA E3 Ubiquitin ligase mediated by *MpCRY* in the liverwort Marchantia polymorpha under blue light. Int. J. Mol. Sci. 23, 158. doi: 10.3390/ijms23010158, PMID: 35008588 PMC8745113

[B46] Zhang G. Q. Xu Q. Bian C. Tsai W. C. Yeh C. M. Liu K. W. (2016). The *Dendrobium catenatum* Lindl. genome sequence in providing insights into polysaccharide synthase, floral development and adaptive evolution. Sci. Rep. 6, 1–10. doi: 10.1038/srep19029, PMID: 26754549 PMC4709516

