# Supplementary material for: Widely targeted metabolomic and transcriptomic analyses of the effects of blue polarized light and ordinary light on Dendrobium officinale
Source: Front Plant Sci. 2026 Feb 24;17:1717555. doi: 10.3389/fpls.2026.1717555 (PMC12971688; doi:10.3389/fpls.2026.1717555)
Supplement: Supplementary file 1 [file Figure S1.pdf › Supplemental information/Figure S1.pdf]

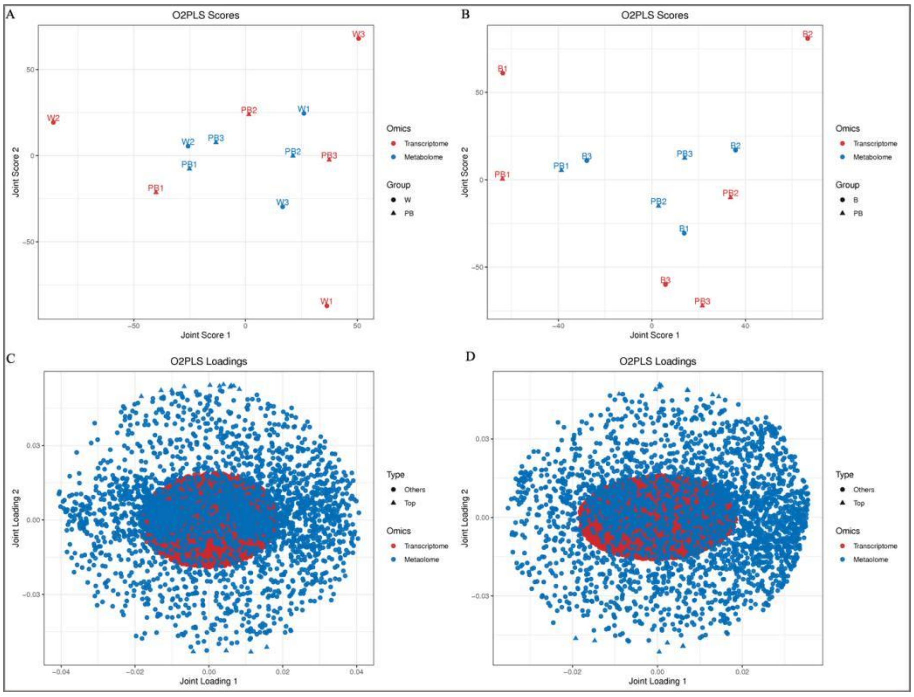

Figure S1. A-B represent O2PLS joint sample score chart in BP-W and BP-B respectively. C-D represent loading diagram of transcriptome and metabolites in O2PLS model in BP-W and BP-B respectively.
